# Drawings of a mental landscape: a peer-led intervention for adolescents in a high school setting

**DOI:** 10.1192/j.eurpsy.2023.672

**Published:** 2023-07-19

**Authors:** A. Barbieri, E. Rossero

**Affiliations:** ^1^Mental Health Department, ASL CN1, Cuneo; ^2^Eclectica+ Research and Training, Turin, Italy

## Abstract

**Introduction:**

Adolescents represent a vulnerable population, with a high prevalence of mental illness and increased levels of subsyndromal psychological distress. Educational settings are central to the lives of young people, and their potentiality to promote mental health is increasingly recognised. The acknowledged role of peer influence on adolescent behaviours indicates peer-led interventions as a promising avenue of youth mental health support.

**Objectives:**

The intervention stems from a pilot called *The Vineyard Project*, which engaged a group of young people with different forms of mental ill-health in local practices of hand-harvesting grape. The pilot was hosted in the region of Langhe (Italy) and was meant to address social anxiety symptoms and poor self-efficacy through the involvement in a culturally meaningful activity within the transformative process of winemaking. The pilot formed the basis of a peer led-intervention in a local Arts high school, aimed to improve mental health knowledge, reduce stigmatising attitudes and promote help-seeking through the mediated connection between students (n = 80) and young people who participated in the *Vineyard Project.*

**Methods:**

Semi-structured interviews with young people participating in the pilot have been conducted and audio-recorded. Interviews explored their experience in the vineyard and its relation with their personal story and the mental health challenges they have been facing. Following a preparatory work with high school teachers, recordings have been anonymized and shared with students to become the object of an art-based workshop.

**Results:**

The practical purpose of the workshop with Arts students was to draw wine labels inspired by their peers’ narratives as they were recorded during interviews. This activity had a double objective: i) to stimulate the ability to listen and foster connection with the experiences shared by young people participating in the vineyard activities; ii) to auction wine bottles labelled by the students to provide financial support for new projects for young people. Feedbacks gathered with students and members of the education community showed that stories shared by participants were considered relatable, experience-near and close to the difficulties that students were familiar with. Consistently with scientific literature on peer support in youth mental health, the intervention showed beneficial effects on the interviewees as well: the opportunity to share their story, making it available to other adolescents who could learn from it and take the project further, stimulated feelings of self-acceptance, personal growth and sense of value.

**Conclusions:**

Emerging results from *the Vineyard Project* suggest that a dialogue between peers, undertook in a non-medicalised framework, can foster connection and empathy, breaking down taboos about mental health, reducing self-stigma and eventually increasing help-seeking intentions.

**Disclosure of Interest:**

None Declared

